# Assessment of Ultrasound Utilization for Peripheral Venous Access in Nursing Practices. An Observational Study From an Italian University Hospital

**DOI:** 10.37825/2239-9747.1053

**Published:** 2024-07-18

**Authors:** Valentina Cerrone, Vincenzo Andretta, Angela Prendin, Noemi Romano, Veronica Strini, Luigi Fortino, Biagio Santella, Giovanni Boccia

**Affiliations:** a Oncology Unit, “San Giovanni di Dio e Ruggi d’Aragona” University Hospital, Salerno, Italy; b Department of Medicine, Surgery and Dentistry “Scuola Medica Salernitana”, University of Salerno, Baronissi, SA, Italy; c Palliative Care and Antalgic Therapy/Pediatric Hospice, University Hospital of Padua, Padua, Italy; d Clinical Research Unit, University Hospital of Padua, Padua, Italy

**Keywords:** Nursing, Ultrasound, Peripheral venous access, Difficult venous access

## Abstract

**Background:**

Establishing peripheral intravenous access can be challenging, often resulting in care delays, increased complications, and higher healthcare costs. While ultrasound-guided techniques have shown potential in improving success rates and reducing complications, their utilization by nurses varies significantly.

**Aim:**

This study aims to evaluate the efficacy and utilization of ultrasound for difficult peripheral venous access among nurses at an Italian university hospital.

**Methods:**

Data were collected over six months from 64 nurses across various units. The study assessed the prevalence of ultrasound training, additional education, and the frequency of ultrasound use for venipuncture and tip navigation.

**Results:**

Of the 64 nurses, 60.9% had received prior ultrasound training, with 50% undergoing further education. Despite this, only 50% regularly used ultrasound for venipuncture, and a mere 26.6% employed it for tip navigation.

**Conclusion:**

Enhanced education and training are essential for increasing the utilization of ultrasound techniques among nurses. This, in turn, can optimize patient outcomes and enhance safety in clinical settings.

## 1. Introduction

Peripheral venous access is a routine and essential procedure performed in approximately 80% of hospitalized patients [1]. However, obtaining peripheral intravenous access can be challenging in certain cases, leading to delays in patient care, increased risks of complications, and higher healthcare costs [2,3]. Difficult venous access (DIVA), characterized by two failed attempts at cannulation using visualization and palpation as reference techniques, poses significant challenges in clinical practice [4]. Various factors contribute to DIVA, including patient characteristics such as obesity, peripheral edema, and hypovolemia, as well as previous history of DIVA [5,6].

In recent years, ultrasound has emerged as a valuable tool for improving the success rate of peripheral venous access and reducing associated complications. Ultrasound-guided peripheral venous cannulation provides real-time visualization of veins, enabling precise needle placement and reducing the risk of complications such as arterial puncture, hematoma formation, and catheter misplacement [7,8]. Studies have demonstrated that ultrasound-guided techniques significantly decrease execution times and increase success rates compared to traditional blind techniques [4,9].

Despite the growing body of evidence supporting the efficacy of ultrasound in improving peripheral venous access, its utilization and impact on patient outcomes among healthcare professionals, particularly nurses, remain areas of interest and investigation. Nurses play a vital role in peripheral venous access procedures, and their proficiency in ultrasound-guided techniques can have a significant impact on patient care and safety [10]. Therefore, understanding the utilization patterns, training needs, and barriers to the adoption of ultrasound among nurses is essential for optimizing its implementation and maximizing its benefits in clinical practice.

On these bases, this study aims to assess the success rate and utilization patterns of ultrasound for difficult peripheral venous access among nurses. It also evaluates the educational background and experience level of participating nurses to identify factors influencing ultrasound utilization in nursing practice. By conducting a survey and analyzing the responses of nurses from various healthcare areas, this research seeks to evaluate the extent of ultrasound utilization, identify factors influencing its adoption, and explore its implications for patient care.

## 2. Methods

### 2.1. Study design

Our study design was structured as a survey following STROBE guidelines (Table A1 - see Appendix) to assess ultrasound efficacy and utilization for challenging peripheral venous access among nurses at the “San Giovanni di Dio e Ruggi D'Aragona ” University Hospital of Salerno. The target population comprises all nurses employed at the hospital. The study sample included nurses aged between 26 and 56, with work experience ranging from 2 to 35 years, across various healthcare areas: medical, critical, surgical, onco-hematological, emergency/urgency, and maternal/infantile. Units that were not directly involved in managing vascular access were excluded from the study. The study was conducted over the six months of May–November 2023. Enrollment was carried out over the months of October and November 2023.

### 2.2. Data collection

Data is collected by administering a questionnaire (Table 1) to nurses at the “San Giovanni di Dio e Ruggi d'Aragona” University-Hospital of Salerno, which has been developed and verified by professionals in Nursing and Midwifery Sciences.

The study, after the approval of the General Director of the “San Giovanni di Dio e Ruggi d'Aragona ” University-Hospital of Salerno (prot. n. 24018/2023), was presented to the nursing coordinators of all the Units of the hospital, upon invitation of the Principal Investigator. A quantitative data analysis was conducted using descriptive statistical methods. The data were collected using Excel and all statistical analyses were conducted with the use of STATA SE version 18 [11].

## 3. Results

### 3.1. Sample characteristics

Data collection was conducted among 64 nurses from the “San Giovanni di Dio e Ruggi D'Aragona” University Hospital of Salerno, aged between 26 and 60 years (mean age 40.17 ± 9.86). The enrollment period spanned over 30 days, occurring in the months of October and November 2023. Among the participants, 34.4% were male (n = 22), while 65.6% were female (n = 42) (Table 2).

### 3.2. Nursing experience distribution

**Fig. 1:**
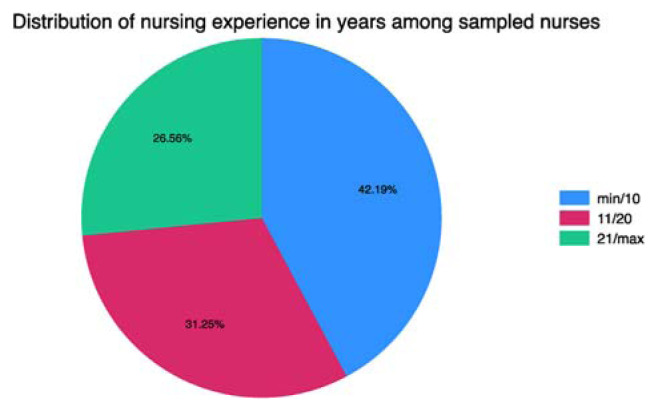
Nursing experience distribution divided into three classes: blue 2–10 years; fuxia 11–20 years; green 21–35 years.

Regarding nurses' experience, it was found that 42.19% of the sample had between 2 and 10 years of experience, 31.25% had between 11 and 20 years of experience, and 26.56% had between 21 and 35 years of experience. The minimum years of experience reported were 2 years, while the maximum was 35 years (Fig. 1).

### 3.3. Educational background

**Fig. 2:**
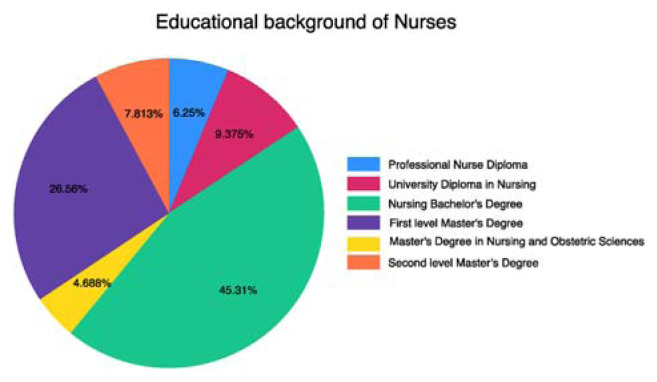
The educational background of nurses included in the study, from the smallest to the highest level.

The educational history of the nurses in the sample was diverse, showing a variety of qualifications and expertise (Fig. 2). Out of the attendees, 6.25% had a University Diploma in Nursing, while 9.38% had a Professional Nurse Diploma. A large portion of the participants, totaling 45.31%, possessed a Nursing Bachelor's Degree, demonstrating a solid educational background in the field. Additionally, 26.56% of individuals had finished a First-level master's degree, indicating continuous learning and growth in the nursing field. Afterward, 4.69% of the nurses had achieved a Master's Degree in Nursing and Obstetric Sciences, indicating increased specialization and knowledge acquisition. Furthermore, 7.81% of individuals opted to pursue a Second-level master's degree. The wide range of educational experiences shows the variety of skills among the surveyed nurses and emphasizes the significance of ongoing education in nursing.

### 3.4. Ultrasound utilization in nursing practice

The study revealed a high level of education and training among the sampled nurses in utilizing ultrasound in nursing practice. Ultrasound was found to be extensively used across various clinical scenarios, including:

- Assessment of venous and arterial heritage
- Facilitation of peripheral venous and arterial access procedures
- Evaluation of the presence of bladder pathology
- Verification of nasogastric tube positioning
- Performance of emergency procedures such as Eco fast
- Assessment of the radial artery
- Assistance in bladder catheter placement

### 3.5. Utilization of vascular ultrasound

Regarding vascular ultrasound specifically, 50% of nurses reported using ultrasound-guided venipuncture, indicating a significant reliance on this technique. Additionally, 26.6% of the sample reported utilizing tip navigation to verify the correct positioning of the catheter. Notably, 57.8% of nurses reported having a dedicated team (comprising nurses or doctors) focused exclusively on acquiring ultrasound-guided vascular access.

### 3.6. Training and knowledge acquisition

**Fig. 3:**
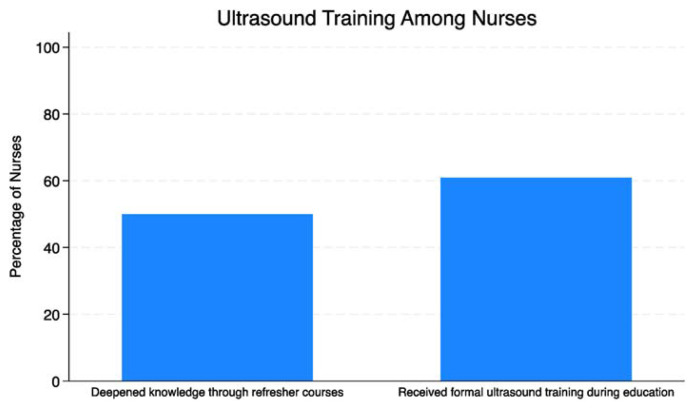
Percentage of nurses training and knowledge acquisition on ultrasound.

The study highlighted the importance of formal training and knowledge acquisition in ultrasound utilization. Approximately 60.9% of the sample had received formal ultrasound training during their education, with an additional 50% of nurses deepening their knowledge through refresher courses (Fig. 3). This emphasis on training underscores the significance of ongoing education in enhancing nursing practice.

### 3.7. Comparison of ultrasound-guided and palpation techniques

**Fig. 4:**
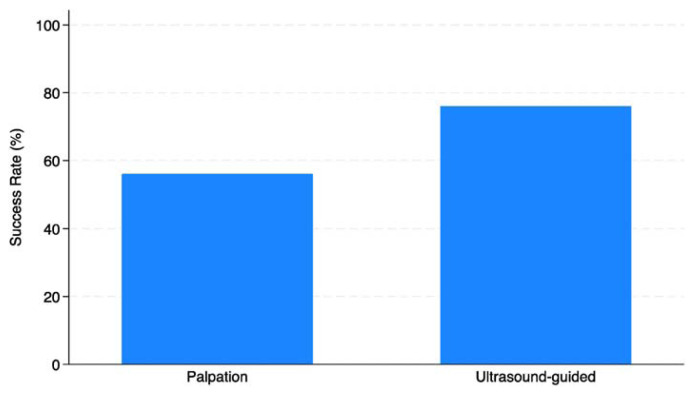
Comparison of success rate between ultrasound-guided and palpation techniques.

Significantly, the study compared the success rates of the ultrasound-guided technique versus the palpation technique in patients with difficult vascular access. The results indicated a substantial difference in success rates, with the ultrasound-guided technique yielding a success rate of 76%, compared to 56% with the palpation technique (Fig. 4). This finding suggests the superiority of ultrasound-guided techniques in challenging vascular access scenarios.

### 3.8. Vascular access team

**Fig. 5:**
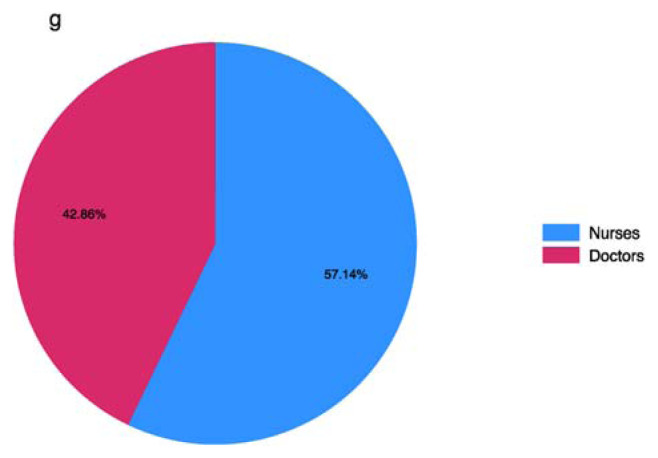
Percentage of nurses and doctors in vascular access team.

In our study, we found that among the nurses surveyed, 42.86% reported the presence of doctors in the vascular access team, while 54.14% reported the presence of nurses (Fig. 5). This indicates a significant involvement of both doctors and nurses in vascular access teams, with nurses comprising the majority.

This distribution reflects the collaborative approach to healthcare delivery, where multiple healthcare professionals work together to ensure optimal patient care [1–3].

The presence of doctors in vascular access teams may contribute additional expertise and support to nurses, potentially enhancing procedural outcomes and patient safety.

Moreover, the predominant presence of nurses underscores their crucial role in vascular access management, highlighting their expertise and proficiency in this area. Collaborative teamwork between doctors and nurses in vascular access teams is essential for providing comprehensive and high-quality patient care.

### 3.9. Evaluation of the use of ultrasound for peripheral venous access in the nursing department compared to nursing education

Ultrasound is used in different hospital units and by nurses (46 of 64 total nurses) with varying educational backgrounds providing a detailed insight into its significance and acceptance in a wide range of clinical settings (Table 3). Out of 9 nurses in the Surgical Area, 5 mentioned that they use ultrasound. Out of these, three nurses possessed a Bachelor's Degree in Nursing. Within the Critical Area, 12 out of 25 nurses made use of ultrasound. The majority of nurses in this region held a Nursing Bachelor's Degree, with 7 nurses, while 4 nurses had a Professional Nurse Diploma. On the flip side, in the Emergency/Urgency Area, 3 out of 5 nurses mentioned using ultrasound, while 2 nurses had a Professional Nurse Diploma. In all departments, every nurse in the Surgical Wing mentioned using ultrasound, but none of the nurses in the Maternal/Infant Area reported using ultrasound. These number-based results highlight the importance of considering the unique circumstances of each unit and the educational backgrounds of nurses when evaluating the incorporation of ultrasound in nursing practice, indicating the significance of customized education and resource distribution in various clinical environments.

## 4. Discussion

The results of our study revealed a substantial difference in success rates between ultrasound-guided and palpation techniques for challenging peripheral venous access scenarios among nurses at the “San Giovanni di Dio e Ruggi D'Aragona” University Hospital of Salerno. Ultrasound-guided techniques demonstrated superior outcomes, highlighting their efficacy in improving procedural efficiency and patient outcomes [21–24]. This finding aligns with previous research conducted in Italy and internationally, which has consistently demonstrated the effectiveness of ultrasound-guided techniques in improving success rates and reducing complications associated with venous access procedures [1–5].

Similar to the study by Barone et al. [12], our findings underscore the benefits of ultrasound guidance in reducing the number of attempts required for successful cannulation, thereby enhancing patient satisfaction, and decreasing procedural complications. Additionally, our results corroborate the findings of Romagnoli et al. [13],who emphasized the importance of ultrasound guidance in patients with challenging venous access, showing a notable increase in success rates compared to traditional palpation techniques. Internationally, studies by Lamperti et al. [14] and Shokoohi et al. [15] have highlighted the widespread adoption of ultrasound-guided vascular access procedures across various healthcare settings. These studies have emphasized the role of ultrasound in enhancing procedural outcomes, particularly in challenging patient populations.

Furthermore, research by Fields et al. [4] emphasized the utility of ultrasound in improving first-pass success rates and reducing catheter-related complications, aligning with the findings of the current study. However, the significance of collaboration between doctors and nurses within vascular access teams, emphasizes their complementary roles in optimizing patient care. Doctors bring advanced medical expertise and decision-making skills to the team, while nurses contribute specialized skills in venous access procedures. This collaborative approach ensures comprehensive patient care, minimizing complications and optimizing procedural outcomes [16]. Effective interdisciplinary communication and teamwork promote knowledge exchange, care coordination, and the implementation of best practices, ultimately enhancing patient-centered care and improving patient outcomes [1,7].

Our research adds to the current body of knowledge by providing information on the schooling and preparation of nurses in using ultrasound, presenting an understanding of its use in different clinical environments and among nurses with different educational levels [17–20]. The rise in ultrasound use in critical care shows a growing need for advanced vascular access methods, underscoring the importance of specialized training and support in these units to address changing clinical requirements.

Our results emphasize the crucial importance of organized instruction in aiding the incorporation of new technologies such as ultrasound in nursing practice. Nurses who have completed a Bachelor's Degree in Nursing show increased levels of ultrasound usage, highlighting the impact of further education in accepting and successfully integrating new methods of patient care. This highlights the significance of ongoing professional growth and gaining specific skills to improve nursing practice and maximize patient care results. Additionally, our research underscores differences in the use of ultrasound among various clinical departments, especially in departments such as Maternal/Infantile where there is a lack of ultrasound utilization. To tackle these differences, specific educational efforts, distribution of resources, and cooperation between different fields may be needed to guarantee the fair availability of advanced technologies and enhance patient results in various healthcare environments.

In general, our results show an increasing acknowledgment of ultrasound's importance in improving procedural efficiency and patient safety in nursing practice.

Moreover, our study compared the success rates of ultrasound-guided versus palpation techniques in challenging vascular access scenarios. The results indicated a substantial difference in success rates, with ultrasound-guided techniques yielding superior outcomes. This finding underscores the importance of ultrasound guidance in improving procedural efficiency and patient outcomes [21–24].

The findings of this study hold significant clinical relevance in the realm of patient care, particularly in the context of improving outcomes related to peripheral venous access procedures. Difficulty in obtaining peripheral intravenous access poses considerable challenges in clinical practice, often leading to delays in patient care, increased risks of complications, and higher healthcare costs [25,26]. By evaluating the success rates and utilization patterns of ultrasound for difficult peripheral venous access among nurses, this study provides valuable insights that can directly impact patient safety and procedural efficiency [27]. Ultrasound-guided techniques offer real-time visualization of veins, enabling precise needle placement and reducing the risk of complications such as arterial puncture, hematoma formation, and catheter misplacement [28,29]. These benefits translate into enhanced patient safety, minimized procedural complications, and improved overall healthcare delivery [30].

One of the paramount concerns in healthcare practice is patient safety, and the utilization of ultrasound-guided techniques for peripheral venous access plays a crucial role in mitigating risks and ensuring optimal patient outcomes [31]. By minimizing the incidence of complications associated with traditional blind techniques, such as accidental arterial puncture or catheter misplacement, ultrasound-guided procedures contribute significantly to patient safety [32]. Moreover, the ability to visualize veins in real-time enhances procedural accuracy and reduces the likelihood of repeated attempts, thereby minimizing patient discomfort and enhancing overall satisfaction with care [33]. The implementation of ultrasound-guided techniques as a standard practice can therefore have profound implications for patient safety and quality of care [34]. In addition to improving patient outcomes, ultrasound-guided procedures have the potential to yield cost savings for healthcare institutions by reducing procedure times, decreasing complication rates, and improving resource utilization [35].

While the initial investment in ultrasound equipment and training may incur some upfront costs, the long-term benefits in terms of reduced procedural complications and improved efficiency can result in significant cost savings [36]. Studies have shown that ultrasound-guided techniques are associated with higher success rates and lower complication rates compared to blind techniques, leading to reduced healthcare costs and resource utilization [37–40]. By optimizing procedural efficiency and minimizing the need for repeat attempts, ultrasound-guided procedures contribute to streamlined workflows, reduced staff workload, and enhanced overall productivity [41–45].

### 4.1. Limitations of the study

While this study provides valuable insights into the utilization of ultrasound-guided techniques among nurses in a specific hospital setting, there are certain limitations to consider. Firstly, the study was conducted at a single institution, which may limit the generalizability of the findings to other healthcare settings. Additionally, the sample size was relatively small, which could affect the statistical power and precision of the results. Furthermore, the study relied on self-reported data, which may be subject to recall bias or social desirability bias. Future research with larger sample sizes and multi-center studies could provide a more comprehensive understanding of ultrasound utilization among nurses.

### 4.2. Implications for future research

Future research endeavors in the field of ultrasound-guided vascular access procedures hold immense potential for advancing patient care and healthcare delivery. By addressing key areas of inquiry, such as the impact on long-term patient outcomes, cost-effectiveness, and standardization of training protocols, researchers can build upon the findings of this study and contribute to a deeper understanding of ultrasound utilization in nursing practice [46–50]. Moreover, longitudinal studies are warranted to assess the sustained benefits of ultrasound training on nursing practice beyond the immediate post-training period [47]. These studies can provide insights into the long-term impact of ultrasound-guided techniques on procedural outcomes, patient safety, and healthcare resource utilization. Additionally, comparative studies across different healthcare settings can further evaluate the cost-effectiveness of ultrasound-guided procedures compared to traditional blind techniques [48]. Such studies can inform healthcare decision-makers and stakeholders about the economic implications of adopting ultrasound-guided techniques in clinical practice. Furthermore, research focusing on the development and implementation of standardized protocols and guidelines for ultrasound training in nursing education curricula is essential [49]. Standardized training programs can ensure consistency in ultrasound education and competency among nursing students, thereby enhancing procedural efficiency and patient safety. For this aim gamification strategies and artificial intelligence-based methods can be adopted [51,52]. Importantly, by integrating ultrasound training into the nursing education curriculum, institutions can prepare future nurses to effectively utilize ultrasound-guided techniques in clinical practice.

## 5. Conclusions

In conclusion, our study provides valuable insights into the utilization of ultrasound for difficult peripheral venous access among nurses at the “San Giovanni di Dio e Ruggi D'Aragona” University Hospital of Salerno. The findings underscore the importance of ultrasound guidance in improving success rates and reducing complications associated with venous access procedures. Additionally, the study highlights the significant role of nurses in ultrasound utilization, emphasizing the importance of education, training, and interdisciplinary collaboration in optimizing procedural outcomes and enhancing patient safety. By identifying factors influencing ultrasound utilization and assessing its impact on patient care, this research contributes to the ongoing efforts to enhance healthcare delivery and improve patient outcomes.

## Figures and Tables

**Table 1:** Questionnaire items administered to nurses for assessing ultrasound utilization in peripheral venous access procedures.

Questionnaire on assessment of ultrasound utilization in intravenous vascular access among nursing staff						
1. Age						
2. Gender	M	F				
3. What is your highest level of education completed in the field of nursing?	Professional Nursing Diploma	University Diploma	Bachelor's Degree (3-Year)	Master's Degree (I)	Master's degree in nursing and Midwifery Sciences	Master's Degree (II)
4. What is your primary work area or department within the healthcare facility?	Onco-hematological	Emergency/Urgency	Critical Area	Surgical Area	Maternal/Infantile	
5. How many years have you been working as a nurse?						
6. Have you received training or education in utilizing ultrasound technology?	Yes	Not				
7. Have you had the opportunity to attend refresher courses related to nursing ultrasound, specifically as an operational tool for obtaining difficult venous access?	Yes	Not				
8. Do you have the opportunity to utilize ultrasound as an adjunct during nursing care in your current work environment?	Yes	Not				
9. When is ultrasound typically utilized in your nursing practice?	Venous/Arterial Asset Evaluation	Verification of Nasogastric Tube Positioning	Evaluation of Bladder Distension	Peripheral Venous/Arterial Access Retrieval	Catheter Placement Verification	Radial Artery Cannulation
10. Do you utilize ultrasound for venous puncture during nursing care procedures?	Yes	Not				
11. Do you utilize ultrasound for tip navigation during vascular access procedures?	Yes	Not				
12. Is there a specific team within your department solely dedicated to utilizing ultrasound for obtaining central venous access?	Yes	Not				
13. Which professionals are involved in the dedicated ultrasound team for obtaining central venous access in the department where you work?	Nurses	Doctors				
14. Is there a dedicated nursing team specifically tasked with managing vascular access procedures?	Yes	Not				

**Table 2:** Demographic characteristics of participants, showing mean age, gender distribution, and age range.

Variables	Mean ± SD	N (%)	Range
**Age**	40.17 ± 9.89		26–60
**Gender**			
Men		22 (34.4)	
Women		42 (65.6)	

**Table 3:** Ultrasound usage for peripheral venous access in nursing department compared to nurse education.

Department	Professional Nurse Diploma	University Diploma in Nursing	Nursing Bachelor's Degree	First Level Master's Degree	Master's Degree in Nursing and Obstetric Sciences	Second Level Master's Degree	Total
Surgical Area	0	1	5	3	0	0	9
Critical Area	0	4	12	7	1	1	25
Medical Area	0	0	0	0	0	2	2
Oncology/Hematology	1	0	2	1	0	1	5
Emergency/Urgency Area	0	1	3	1	0	0	5
Maternal/Infantile Area	0	0	0	0	0	0	0
Total	1	6	22	12	1	4	46

## Data Availability

Not applicable.

## Appendix

**Table A1:** STROBE Statement - Checklist of items that should be included in reports of observational studies.

STROBE Statement - Checklist of items that should be included in reports of observational studies		
	Item No	Recommendation
**Title and abstract**	1	(*a*) Assessment of Ultrasound Utilization for Peripheral Venous Access in Nursing Practices. An observational study from an Italian University-Hospital (*b*) Background: Establishing peripheral intravenous access can be challenging, often resulting in care delays, increased complications, and higher healthcare costs. While ultrasound-guided techniques have shown potential in improving success rates and reducing complications, their utilization by nurses varies significantly. Aim: This study aims to evaluate the efficacy and utilization of ultrasound for difficult peripheral venous access among nurses at an Italian university hospital. Methods: Data were collected over six months from 64 nurses across various units. The study assessed the prevalence of ultrasound training, additional education, and the frequency of ultrasound use for venipuncture and tip navigation. Results: Of the 64 nurses, 60.9% had received prior ultrasound training, with 50% undergoing further education. Despite this, only 50% regularly used ultrasound for venipuncture, and a mere 26.6% employed it for tip navigation. Conclusion: Enhanced education and training are essential for increasing the utilization of ultrasound techniques among nurses. This, in turn, can optimize patient outcomes and enhance safety in clinical settings.
**Introduction**		
Background/rationale	2	Peripheral venous access is a routine procedure in hospitals, but difficulties can arise, leading to delays, complications, and increased costs. Difficult venous access (DIVA), defined by two failed cannulation attempts, presents challenges. Factors such as patient characteristics and previous DIVA history contribute to the challenge. Ultrasound has emerged as a valuable tool to improve success rates and reduce complications. Real-time visualization provided by ultrasound facilitates precise needle placement, reducing risks. Studies have shown significant benefits of ultrasound-guided techniques over traditional blind techniques. However, the utilization and impact of ultrasound among nurses remain areas of investigation, given their vital role in peripheral venous access procedures.
Objectives	3	The study aims to evaluate the efficacy and utilization patterns of ultrasound for difficult peripheral venous access among nurses in an Italian University Hospital. Specifically, the objectives are: - To assess the success rate of ultrasound-guided techniques for difficult peripheral venous access among nurses. - To explore the utilization patterns of ultrasound among nurses across different healthcare areas. - To evaluate the educational background and experience level of participating nurses to identify factors influencing ultrasound utilization. - To investigate the presence of dedicated teams for ultrasound-guided vascular access and the professionists involved. - To contribute to the existing knowledge on peripheral venous access and inform strategies for improving patient outcomes and healthcare delivery.
**Methods**		
Study design	4	A survey to assess efficacy of ultrasound for difficult peripheral venous access among nurses at San Giovanni di Dio e Ruggi D'Aragona University Hospital, Salerno.
Setting	5	The study was conducted at San Giovanni di Dio e Ruggi D'Aragona University Hospital, located in Salerno, Italy. This hospital serves as a tertiary care center and encompasses various healthcare areas, including medical, surgical, critical, onco-hematological, emergency/urgency, maternal/infantile departments.
Participants	6	The study sample comprised 64 nurses employed at San Giovanni di Dio e Ruggi D'Aragona University Hospital. Nurses from different healthcare areas within the hospital were included in the sample, providing a diverse representation of nursing practice.
Variables	7	Independent variables: Nurses' educational background, years of experience, training in ultrasound, and utilization of ultrasound for difficult peripheral venous access. Dependent variables: Success rates of ultrasound-guided techniques for venipuncture and catheter positioning, presence of dedicated teams for ultrasound-guided vascular access.
Data sources/measurement	8	Data were collected through a structured questionnaire administered to the participating nurses. The questionnaire was developed using the Delphi method and underwent content validation to ensure relevance and comprehensiveness. The responses obtained from the questionnaire served as the primary data source for the study.
Bias	9	Potential sources of bias in the study include self-reporting bias, recall bias, and social desirability bias, as data were collected through self-administered questionnaires. Efforts were made to minimize bias through the use of validated questionnaire items and by ensuring confidentiality and anonymity in data collection.
Study size	10	The study included a total of 64 nurses from San Giovanni di Dio e Ruggi D'Aragona University Hospital, providing a sufficient sample size to conduct meaningful analyses and draw reliable conclusions regarding ultrasound utilization for peripheral venous access among nurses.
Quantitative variables	11	Quantitative variables assessed in the study included nurses' years of experience, success rates of ultrasound-guided venipuncture and catheter positioning, and the proportion of nurses receiving ultrasound training and utilizing ultrasound for venous access procedures.
Statistical methods	12	Descriptive statistical methods were employed to analyze the collected data, including measures of central tendency (mean, median) and dispersion (standard deviation, range). Data were analyzed using Excel for initial data management and STATA SE version 18 for statistical analyses. The results were presented using tables, figures, and descriptive summaries to provide a comprehensive overview of the study findings.
**Results**		
Participants	13	(a) The participants in this study comprised 64 nurses employed at San Giovanni di Dio e Ruggi D'Aragona University Hospital in Salerno, Italy. These nurses represented various healthcare areas within the hospital, including medical, surgical, critical, onco-hematological, emergency/urgency, maternal/infantile departments.
Descriptive data	14	(a) **Demographic Characteristics**: The participants' age ranged from 26 to 60 years, with a mean age of 40.17 years (± 9.86 SD). Among the participants, 34.4% were male (n = 22), while 65.6% were female (n = 42). **Nursing Experience Distribution**: The distribution of nursing experience showed that 42.19% of participants had between 0 and 10 years of experience, 31.25% had between 11 and 20 years, and 26.56% had between 21 and 35 years of experience. The minimum years of experience reported was 2 years, while the maximum was 35 years. **Educational Background**: The educational background of the nurses varied, with 6.25% holding a University Diploma in Nursing, 9.38% possessing a Professional Nurse Diploma, 45.31% holding a bachelor's degree in nursing, 4.69% having a master's degree in nursing and Obstetric Sciences, 26.56% completing a First level master's degree, and 7.81% pursuing a Second level master's degree.
Main results	16	(*a*) **Ultrasound Utilization in Nursing Practice**: The study revealed a high level of education and training among nurses in utilizing ultrasound in nursing practice. Ultrasound was extensively used for various clinical purposes, including venous and arterial assessment, peripheral access procedures, bladder pathology evaluation, nasogastric tube positioning verification, emergency procedures, radial artery assessment, and bladder catheter placement assistance. **Utilization of Vascular Ultrasound**: Specifically focusing on vascular ultrasound, 50% of nurses reported using ultrasound-guided venipuncture, while 26.6% utilized tip navigation to verify catheter positioning. Furthermore, 57.8% of nurses reported the presence of dedicated teams for ultrasound-guided vascular access, comprising nurses and/or doctors. **Training and Knowledge Acquisition**: Approximately 60.9% of nurses had received formal ultrasound training during their education, with an additional 50% enhancing their knowledge through refresher courses. This emphasis on training highlights the significance of continuous education in improving nursing practice.
Other analyses	17	**Comparison of Ultrasound-Guided and Palpation Techniques**: The study compared the success rates of ultrasound-guided techniques versus traditional palpation techniques for difficult vascular access. Ultrasound-guided techniques demonstrated a higher success rate (76%) compared to palpation techniques (56%), emphasizing the superiority of ultrasound guidance in challenging scenarios. **Vascular Access Team Composition**: Among the surveyed nurses, 42.86% reported the presence of doctors in the vascular access team, while 54.14% reported the presence of nurses. This distribution indicates significant involvement of both professions in vascular access teams, highlighting their complementary roles in optimizing patient care.
**Discussion**		
Key results	18	**Ultrasound Utilization**: The study highlighted a high level of ultrasound utilization among nurses, with the majority utilizing ultrasound for various clinical purposes, including venous and arterial assessment, peripheral access procedures, and emergency interventions. Specifically, 50% of nurses reported using ultrasound-guided venipuncture, indicating a significant reliance on this technique. **Training and Knowledge Acquisition**: A noteworthy finding was the emphasis on formal ultrasound training among nurses, with approximately 60.9% having received formal training during their education. Moreover, 50% of nurses further enhanced their knowledge through refresher courses, highlighting the importance of continuous education in optimizing nursing practice. **Comparison of Techniques**: The study compared the success rates of ultrasound-guided techniques versus traditional palpation techniques for difficult vascular access. Ultrasound-guided techniques demonstrated a higher success rate (76%) compared to palpation techniques (56%), underscoring the superiority of ultrasound guidance in challenging scenarios.
Limitations	19	**Single-Center Study**: The study was conducted at a single institution, which may limit the generalizability of the findings to other healthcare settings. Replication of the study across multiple centers could enhance the external validity of the results. **Small Sample Size**: The sample size of 64 nurses may limit the statistical power and precision of the results. Future studies with larger sample sizes could provide more robust evidence.
Interpretation	20	The findings suggest that ultrasound-guided techniques are widely utilized among nurses in clinical practice and are associated with higher success rates compared to traditional palpation techniques. Moreover, the emphasis on formal training underscores the importance of continuous education in enhancing nursing practice and patient outcomes.
Generalizability	21	While the study was conducted at a single institution, the findings provide valuable insights into ultrasound utilization among nurses in a hospital setting. However, further research across multiple healthcare settings and with larger sample sizes is warranted to enhance the generalizability of the results.
**Other information**		
Funding	22	Not applicable.
